# Quality of orthodontic care—A multicenter cohort study in a German convenience sample: Part 2: Cross-sectional patient-reported outcomes after orthodontic treatment

**DOI:** 10.1007/s00056-024-00528-z

**Published:** 2024-04-26

**Authors:** Isabelle Graf, Niko Christian Bock, Theodosia Bartzela, Vera Röper, Uwe Schumann, Karl Reck, Hans-Joachim Helms, Karolin Hoefer, Ulrike Fritz, Michael Wolf, Dirk Wiechmann, Paul-Georg Jost-Brinkmann, Sabine Ruf, Bert Braumann

**Affiliations:** 1 Department of Orthodontics, Faculty of Medicine and University Hospital of Cologne, University of Cologne, Kerpener Str. 32, 50931 Cologne, Germany; 2 Department of Orthodontics, Faculty of Medicine and University Hospital of Gießen, University of Gießen, Gießen, Germany; 3 Department of Orthodontics, Faculty of Medicine Carl Gustav Carus, Technische Universität Dresden, Dresden, Germany; 4 Department of Orthodontics, Hannover Medical School, Hannover, Germany; 5 Orthodontic practice, Essen, Germany; 6 Orthodontic practice, Pulheim, Germany; 7 Department of Medical Statistics, University Medical Centre, Göttingen, Germany; 8 Department of Operative Dentistry and Periodontology, Faculty of Medicine and University Hospital of Cologne, University of Cologne, Cologne, Germany; 9 Department of Orthodontics, Faculty of Medicine and University Hospital RWTH Aachen, RWTH Aachen, Aachen, Germany; 10 Center for Dental and Craniofacial Sciences, Department of Orthodontics and Dentofacial Orthopedics, Charité—Universitätsmedizin Berlin, corporate member of Freie Universität Berlin and Humboldt-Universität zu Berlin, Berlin, Germany; 11 Orthodontic practice, Bad Essen, Germany

**Keywords:** Patient centeredness, Oral Health Impact Profile, Treatment outcome, Child Oral Health Impact Profile, Oral health-related quality of life, Patientenorientierung, Oral Health Impact Profile, Behandlungsergebnis, Child Oral Health Impact Profile, Oral health-related quality of life

## Abstract

**Objectives:**

In light of the growing interest in orthodontic care and its effectiveness in Germany, part 2 of this multicenter cohort study evaluated patient-reported outcomes such as oral health-related quality of life (OHRQoL), oral hygiene habits, oral health beliefs, and potential influencing factors.

**Methods:**

Of 586 patients screened from seven German study centers, data from 343 patients were analyzed for this part of the study. At the end of their orthodontic treatment, study participants filled out a questionnaire of either the German long version of the Oral Health Impact Profile (OHIP-G 49) or the German short version of the Child Oral Health Impact Profile (COHIP-19), depending on their age, as well as questions about their oral hygiene behavior and beliefs. Patient-, treatment- and occlusion-related factors were analyzed to account for potential influencing factors with regard to patients’ OHRQoL after orthodontic treatment.

**Results:**

In all, 222 study participants filled out the OHIP-based and 121 the COHIP-based questionnaire. The mean OHIP-G 49 score was 12.68 and the mean OHIP-G 14 score was 3.09; the mean COHIP-19 score was 6.52 (inverted score 69.48). For OHIP-G 49 scores, a nonsignificant trend towards a higher score for male patients (14.45 vs 11.54; *p* = 0.061) was detected, while this trend was inverse for the COHIP-19 scores, i.e., female patients reported more impairment (total score 6.99 vs. 5.84; *p* = 0.099). Analyses suggested a trend towards better OHRQoL for patients who classified for the Peer Assessment Rating (PAR) Index improvement rate group ‘greatly improved’ as well as for nonsmokers. Oral hygiene habits and beliefs after orthodontic treatment were estimated to be good.

**Conclusion:**

In this German cohort, OHRQoL proved to be good and was rather unimpaired after orthodontic treatment. Furthermore, self-reported oral hygiene behavior and oral health beliefs represented good health awareness.

**Supplementary Information:**

The online version of this article (10.1007/s00056-024-00528-z) contains supplementary material, which is available to authorized users.

## Abstract (de)

**Ziel:**

Das öffentliche Interesse an kieferorthopädischen Behandlungen und deren Qualität in Deutschland wächst zunehmend. In diesem Zusammenhang war es das Ziel des zweiten Teils dieser multizentrischen Kohortenstudie, „patient-reported outcomes“, wie die mundgesundheitsbezogene Lebensqualität (MLQ), Mundhygieneangewohnheiten und subjektive Mundgesundheitsüberzeugungen, sowie potenzielle Einflussfaktoren nach kieferorthopädischer Behandlung zu erfassen.

**Methoden:**

Von 586 gescreenten Patienten aus 7 deutschen Studienzentren konnten 343 in die Studie einbezogen werden. Ein digitaler Fragebogen wurde von allen Studienteilnehmern am Ende der kieferorthopädischen Retentionszeit ausgefüllt. Dieser beinhaltete entweder die validierten Fragen der deutschen Versionen des Oral Health Impact Profile (OHIP-G 49) bzw. des Child Oral Health Impact Profile (COHIP-19), abhängig vom Alter der Probanden, sowie zusätzlich Fragen zur Mundgesundheit. Patientenindividuelle, behandlungs- und okklusionsbezogene Faktoren wurden hinsichtlich potenzieller Einflussgrößen auf die MLQ nach kieferorthopädischer Behandlung analysiert.

**Ergebnisse:**

Der mittlere OHIP-G-49-Summenwert war 12,68 (*n* = 222), der mittlere OHIP-G-14-Summenwert 3,09, der mittlere COHIP-19-Summenwert 6,52 (invertierter Summenwert 69,48; *n* = 121). In Bezug auf die OHIP-G-49-Summenwerte zeigte sich ein nicht signifikanter Trend zu höheren Summenwerten bei männlichen Probanden (14,45 vs. 11,54; *p* = 0,061). Für die COHIP-19-Summenwerte war dieser Trend umgekehrt, d. h. Probandinnen berichteten mehr Einschränkungen der MLQ (6,99 vs. 5,84; *p* = 0,099). Weitere Analysen stellten einen Trend zu einer besseren MLQ bei Patienten der „greatly improved“ Gruppe, d. h. bei einer hohen PAR(Peer Assessment Rating)-Index-Verbesserung, heraus; die MLQ war ebenfalls weniger eingeschränkt bei Nichtrauchern. Die Mundgesundheit in Bezug auf die Mundhygieneangewohnheiten und Einstellungen der Patienten nach kieferorthopädischer Behandlung war gut.

**Zusammenfassung:**

Die MLQ dieser deutschen Kohorte war nach kieferorthopädischer Behandlung gut und nahezu uneingeschränkt. Das berichtete Mundgesundheitsverhalten stand für ein gutes Mundgesundheitsbewusstsein der Probanden.

## Introduction

Patient-centered care has become increasingly important in orthodontics. As clinicians we aspire to achieve high-quality treatment outcomes and seek to do this in an evidence-based manner. Part 1 of this German cohort study dealt with orthodontic treatment effectiveness, showing that orthodontic treatments were mostly high-quality treatments and able to significantly improve malocclusions [1]. Yet, within a modern healthcare system, the assessment of the quality of clinical care and its effectiveness should not only focus on occlusal measurements, but must involve patient-reported outcomes. Such patient-based evidence reveals important information about how patients function and feel with regard to their orthodontic treatment. As a multidimensional construct oral health-related quality of life (OHRQoL) can be assessed in order to elucidate patients’ physical and psychosocial well-being [2]. OHRQoL seems to be linked to malocclusion, or to specific dentofacial traits that might impair physical and psychosocial well-being [3]. Among other traits, excessive overjet with incompetent lip closure, a deviating overbite or crowding of anterior teeth have been discussed in this context [4–9]. Correction of these specific malocclusions through orthodontic treatments has been shown to be able to reduce such impairment, leading to better OHRQoL [10, 11]. However, active orthodontic treatment might temporarily reduce the level of OHRQoL, depending on the type of appliance used, the specific treatment stage and the initial malocclusion [12]. Hence, OHRQoL and its relation to malocclusion and/or orthodontic treatment outcomes are crucial pillars of patient-centered, high-quality orthodontic care.

In general, there are several ways to measure OHRQoL of children and adults. Validated patient-reported outcome measures (PROMs) are the Oral Health Impact Profile (OHIP) [13] and the Child Oral Health Impact Profile (COHIP) [14–16]. Multiple items within several psychological, physical and social dimensions lead to a final score that stands for a rather impaired or unimpaired quality of life with regard to oral health. These PROMs have frequently been used in international research, thus, providing a good basis for comparisons between different study groups.

Besides OHRQoL, patient-reported outcomes like oral hygiene habits are of interest in the context of orthodontic treatment [17, 18]. Research has shown that patients might change their oral hygiene habits in the course of orthodontic treatment and the concomitant need for more intense oral hygiene care [19, 20]. The latter is essential in order to keep both soft and hard tissues healthy during treatment with fixed appliances [21]. In addition, it might be important to learn more about patients’ dental awareness and locus of control [22]. Whether one sees an external locus of control with regard to one’s oral condition or rather an internal one might uncover highly interesting factors of one’s sense of coherence or sense of self-efficacy, which consequently might influence orthodontic treatment with regard to adherence to treatment and quality of treatment [23, 24].

Up to now, national research about the above-mentioned topic is sparse. Thus, part 2 of this multicenter cohort study aimed to explore patient-reported outcomes after orthodontic treatment and potential influencing factors within this German convenience sample. Yet, it has to be noted from the beginning on that due to the cross-sectional character of the study, pre-orthodontic PROMs are missing within this study population.

## Subjects and methods

### Study participants

All corresponding ethics committees, the leading one being the Ethics Committee of the University Hospital of Cologne (#14-425), approved this multicenter cohort study. Signed informed consent from patients and/or their legal guardians was mandatory. All patient-related data were pseudonymized. The detailed recruitment procedure used at all study centers had been discussed with the Clinical Trials Center of the University Hospital of Cologne before the study started. Even though recruitment and screening procedures have been described in part 1 of this study [1], a short summary is given here:

As part of the initialization of this multicenter study, the principal investigator (IG) asked all current study centers to participate according to existing research connections and structures. Thus, study center selection was not random. Consequently, this cohort study is based on a convenience sample. Yet, other potential German orthodontic practitioners would have also had the chance to participate as the research project was transparently described prior to study start through various ways (e.g., e‑mail to all delegates of the DGKFO [Deutsche Gesellschaft für Kieferorthopädie e. V.] and announcements at national meetings of university professors) [1].

**Fig. 1:**
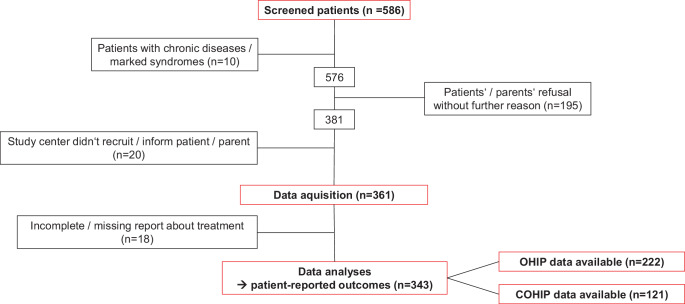
Flow chart of screened, excluded and enrolled patients. *OHIP* Oral Health Impact Profile, *COHIP* Child Oral Health Impact Profile Flussdiagramm aller gescreenten, ausgeschlossenen und in die Studie aufgenommenen Patienten. *OHIP* Oral Health Impact Profile, *COHIP* Child Oral Health Impact Profile

As part of the recruitment procedure, every upcoming posttreatment record was to be screened, resulting in a total of 586 screened patients from seven German study centers (three orthodontic practices and four orthodontic departments of university hospitals). Screening logs as well as repeated monitoring of the recruitment process by the principal investigator (IG) were tools to limit the possibility of patient selection within each study center. There was no significant difference regarding the initial malocclusion severity according to the Peer Assessment Rating (PAR) Index (mean PAR score 25.83 at practices vs. 26.07 at university hospitals, *p* = 0.520). The recruitment period lasted from 5–17 months and did not start simultaneously at all study centers; the ratio between included and screened patients ranged from 31.5–78.4%. The PAR score reduction rate had no influence on this ratio, so the possibility of selection bias *within* each study center should be rather low. Of the screened patients, 361 patients were included in this study. The total sample size comprised all patients with filled out questionnaires. The potential maximum sample size was limited by part 1 of this research project [1]. Inclusion criteria were orthodontically treated patients ≥ 11 years of age at the end of their orthodontic treatment, prior to posttreatment record taking and signed informed consent. Exclusion criteria were severe systemic diseases, immunosuppression and/or craniofacial syndromes. Due to incomplete and/or missing treatment information, 18 patients were excluded from further analyses, leaving data from 343 patients to be analyzed (Fig. 1).

### Assessment of patient-reported outcomes

Within the framework of the clinical part of this study, all study participants filled out an iPad-based questionnaire (iPAD, Apple Inc., Cupertino, CA, USA). This questionnaire consisted of different parts: one part dealt with OHRQoL and, thus, contained questions from the German long version of the OHIP (OHIP‑G 49 + 3 items; supplementary questionnaire 1) [25] or the German short version of the COHIP (19 items; supplementary questionnaire 2) [16], depending on patients’ age. According to the recommendations in relevant literature, the OHIP-based questionnaire was used for patients being 16 years and older, while all younger patients filled out the COHIP-based questionnaire. Both measures for OHRQoL use a five-point Likert scale ranging from “never”/“rarely” to “often”/“very often” and accordingly, score points from 0–4. Consequently, the maximum score for the OHIP long version is 196 and the maximum score for the COHIP short version is 76. According to their original use, a higher OHIP score stands for a more impaired OHRQoL, while a higher COHIP score reflects a better OHRQoL compared to lower scores [14]. For better readability, negatively worded COHIP items were not reversed as suggested by Broder et al. [14]. Thus, within large parts of this publication both OHIP and COHIP scores are directed in the same way. Yet, reversed COHIP scores are additionally provided in the tables in order to allow for a better comparison with literature. For the same reason, OHIP-G 14 scores were calculated from the long version [26, 27].

In addition to items revolving around OHRQoL, the second part of the questionnaire dealt with oral hygiene behavior. Third, patients’ attitudes towards self-efficacy and locus of control were integrated within the digital questionnaire for participants ≥ 16 years. These questions were derived from the 5th German Oral Health Study [28]. Finally, items about patients’ weight and height and their initial reason for orthodontic consultation were also part of the OHIP-based questionnaire (supplementary questionnaires 1 and 2).

In total, 222 patients filled out the OHIP-based questionnaire designed for participants older than 16 years and 121 patients filled out the COHIP-based questionnaire designed for younger ones. The process of filling out the OHIP-based questionnaire took around 15.2 min (± 9.3 min), while the COHIP-based questionnaire took on average 7.6 min (± 5.7 min).

### Treatment characteristics

All study centers provided several treatment characteristics of their patients such as the information about the indication to treat according to German KIG Index (Kiefer-orthopädische Indikationsgruppen) [29], the duration of active treatment and the appliances and modalities used.

### Assessment of occlusal characteristics

Within the scope of part 1 of this multicenter study, pre- (T0) and posttreatment (T1) study models (T0) were analyzed using the PAR index according to the British weighting system [30]. While the principal investigator (IG) was in charge for six of the German study centers, a second calibrated and PAR-certified examiner (NB) measured all study models from the seventh study center due to potential bias resulting from IG’s affiliation. Inter- and intraexaminer reliability for PAR score measurements were excellent [1].

PAR score reduction rate as defined in ‘greatly improved’, ‘improved’ and ‘worse or no different’ had been assessed in order to evaluate the quality of the course of treatments. In addition, final PAR scores of 5 or less were defined as ‘high-quality treatment outcome’ and were used to account for the quality of the final treatment outcomes [1, 30, 31].

### Statistical analyses

To assess the patient characteristics, the questionnaire results including the mean total OHIP and COHIP scores and subscores as well as the treatment-related parameters were analyzed descriptively, using mean ± standard deviation (SD), as well as minimum and maximum values (min–max). To analyze the potentially confounding impact of patient characteristics such as age or gender as well as treatment-related parameters on OHRQoL (i.e., OHIP and COHIP scores), an unpaired t‑test (Welch–Satterthwaite t‑test) was used. The significance level was set to α = 0.05 leading to a *p*-value *p* < 0.05 to be considered statistically significant. In addition, potential influencing treatment- and occlusion-related factors with regard to patients’ OHRQoL after orthodontic treatment have been investigated using either boxplots or scatter plots and linear regression (r correlation coefficient). Further analysis of the effects of patient characteristics and/or OHRQoL items on the patients’ OHRQoL after orthodontic treatment as well as the PAR reduction (absolute and in %) have been analyzed using univariate analysis as well as multivariate analysis of variance (ANOVA) analysis.

Due to the explorative nature of the study, no alpha correction was performed. All statistical analyses were performed with SAS (v9.4, SAS Institute, Cary, NC, USA).

#### Sample size justification

Sample size estimation was based on the primary outcome ‘occlusal change according to the PAR Index and an expected reduction of the total mean PAR score of at least 75%, in accordance with previous studies [11, 32, 33]. The broader range of patient-, practitioner- and treatment-related factors involved in this cohort study were additionally considered [1]. Due to the explorative nature of the study, no formal sample size calculation was performed for this secondary analysis. Instead, all patients fulfilling the inclusion criteria were included for a specific collection interval in order to represent the orthodontic reality in Germany as best as possible. The potential maximum sample size was limited by part 1 of this research project [1] and the total sample size of part 2 comprised all patients with filled out questionnaires. Selection or nonresponder bias could not be completely ruled out.

## Results

### Characteristics of patients and their treatments

In all, 135 patients who filled out the OHIP-based questionnaire were female and 87 were male (60.8 and 39.2%, respectively). The mean age at study recruitment was 20.3 years, whereas the mean age at treatment start had been 16.4 years.

Of all patients who filled out the COHIP-based questionnaire, 71 were female and 50 were male (58.7 and 41.3%, respectively). The average age at study recruitment was 14.6 years and 11.5 years at the beginning of active orthodontic treatment.

Table 1 shows further patient- and treatment-related characteristics.

**Table 1:** Characteristics of study participants according to OHIP- and COHIP-based data Patientencharakteristika für auf OHIP und COHIP basierte Werte

	OHIP	COHIP
*Female*	135 (60.8%)	71 (58.7%)
*Male*	87 (39.2%)	50 (41.3%)
*Age at active treatment start*	16.4 years	11.5 years
*Age at study recruitment*	20.3 years	14.6 years
*Self-reported BMI*	26.7 (SD 11.4)	–
*Orthodontic practice*	102 (46.0%)	45 (37.5%)
*University hospital*	120 (54.0%)	75 (62.5%)
*Mean PAR T0*	26.73 (SD 11.40)	24.65 (SD 9.18)
*Mean PAR T1*	3.40 (SD 2.98)	3.99 (SD 2.82)
*PAR index reduction*		
Worse or no different	1 (0.5%)	2 (1.7%)
Improved	102 (47.9%)	63 (54.8%)
Greatly improved	110 (51.6%)	50 (43.5%)
*High-quality treatment result* (PAR T1 ≤ 5 points)	177 (83.1%)	92 (80%)
*Orthodontic treatment*		
MBA only	158 (73.8%)	63 (54.3%)
MBA plus RA	53 (24.8%)	46 (39.7%)
RA only	–	6 (5.2%)
Missing data	3 (1.4%)	1 (0.8%)
*Mean active treatment duration*	32.9 months	30.1 months

*Mean* mean value, *SD* standard deviation, *BMI* body mass index, *MBA* multibracket appliance, *RA* removable appliance, *OHIP *Oral Health Impact Profile, *COHIP* Child Oral Health Impact Profile, *PAR* Peer Assessment Rating

### Oral health-related quality of life and influencing factors

#### OHIP

The mean OHIP-G 49 score was 12.68 (± 10.64) and the mean OHIP-G 14 score was 3.09 (± 3.42). Regarding sex-related differences concerning the OHIP total and subscores, male patients reported significantly more psychological and physiological disabilities after orthodontic treatment than females (mean subscores 3.15 and 3.61 vs. 2.18 and 2.49; *p* = 0.015 and *p* = 0.029; Table 2).

**Table 2:** OHIP-G 49, OHIP-G 14 and COHIP 19 scores OHIP-G-49-, OHIP-G-14- und COHIP-19-Werte

	*n* (Female/male)	Total mean	Min–Max/SD	Female mean	Male mean	*p*-value^a^ Female vs. male mean
*OHIP dimensions*	(135/87)	–	–	–	–	–
Functional limitations	222	3.05	0.00–12.00/2.57	3.05	3.03	0.960
Physical pain	222	2.06	0.00–7.00/1.80	1.95	2.23	0.258
Psychological disability	222	2.56	0.00–13.00/2.72	*2.18*	*3.15*	*0.015*
Psychological discomfort	222	0.48	0.00–3.00/0.73	0.41	0.59	0.102
Physiological disability	222	2.93	0.00–17.00/3.45	*2.49*	*3.61*	*0.029*
Social disability	222	0.58	0.00–6.00/1.23	0.53	0.67	0.414
Handicap	222	1.03	0.00–10.00/1.73	0.93	1.17	0.337
OHIP-G 49 total score	222	12.68	0.00–53.00/10.64	11.54	14.45	0.061
OHIP-G 14 total score	222	3.09	0.00–18.00/3.42	2.82	3.49	0.172
*COHIP dimensions*	(72/50)	–	–	–	–	–
Social/emotional, school and self-image	122	2.90 (37.10)	0.00–13.00/2.36	3.21	2.46	0.070
Functional well-being	122	1.01 (14.99)	0.00–7.00/1.33	1.11	0.86	0.271
Oral health	122	2.61 (17.39)	0.00–7.00/1.63	2.67	2.52	0.622
COHIP—total score	122	6.52 (69.48)	0.00–20.00/4.07	6.99	5.84	0.099

Inverted COHIP *Total mean* scores in parentheses

*n* sample size, *Total mean* mean score in total, *SD* standard deviation, *Min* minimum value, *Max* maximum value, *Female mean* and *Male mean* mean scores also discriminated for both sexes, *OHIP* Oral Health Impact Profile, *COHIP* Child Oral Health Impact Profile

^a^Welch–Satterthwaite t‑test, values in italics represent statistical significance at 5%

The total OHIP-G 49 score showed only a nonsignificant trend towards a higher score for male patients (14.45 vs. 11.54; *p* = 0.061; Table 2). Looking at gender-specific differences in detail, male patients had started their orthodontic treatment with a slightly higher total PAR score than female patients (27.20 vs. 26.45) as well as a higher overjet-PAR-subscore (11.41 vs. 9.95). Slightly fewer males than females had a ‘high-quality treatment outcome’ with a total PAR score of 5 or less points at the end of treatment (81.5% vs. 84.1%). Orthodontic treatment for males lasted slightly longer than that for female patients (mean months of treatment 34.8 vs. 31.8). No differences were observed between sexes with regard to their age at study recruitment (mean age 20.2 years for males and 20.4 years for females) nor their orthodontic provider (i.e., university hospital vs. orthodontic practice) or specific treatment procedures like the need for orthognathic surgery.

**Fig. 2:**
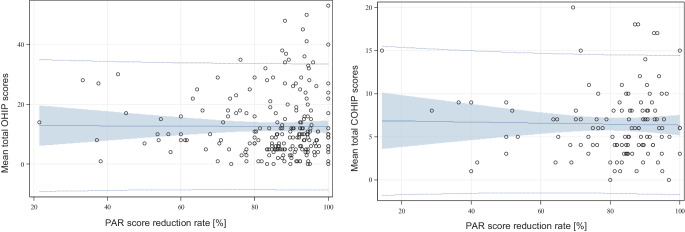
Scatter plot and linear regression analyses for the variables mean OHIP/COHIP score and Peer Assessment Rating (PAR) score reduction rate (%); Pearson’s correlation coefficients −0.007 for OHIP-based data and −0.022 for COHIP-based data; with 95% confidence intervals and prediction limits (*blue* shaded area). *OHIP* Oral Health Impact Profile, *COHIP* Child Oral Health Impact Profile Streudiagramm mit linearen Regressionsanalysen für die Variablen OHIP-/COHIP-Mittelwert und PAR(Peer Assessment Rating)-Index-Reduktionsrate (in %); Pearsons Korrelationskoeffizient −0,007 für OHIP- und −0,022 für COHIP-Werte; 95%-Konfidenzintervalle und Vorhersagegrenzen (*hellblau* getönt). *OHIP* Oral Health Impact Profile, *COHIP* Child Oral Health Impact Profile

Looking at treatment- and occlusion-related aspects as potential influencing factors with regard to patients’ OHRQoL after orthodontic treatment—independent of sex-related differences—neither the PAR score reduction rate (Fig. 2) nor the final PAR score significantly affected OHRQoL. Yet, patients with a “greatly improved” orthodontic treatment result had both a lower mean OHIP-G 49 score and a lower mean OHIP-G 14 score after treatment than patients whose treatment results did not classify for this category (OHIP-G 49 score 11.88 (± 10.32) vs. 13.26 (± 10.94), OHIP-G 14 score 2.85 (± 3.35) vs. 3.16 (± 3.46), respectively; *p* = 0.346 and *p* = 0.520; Table 3).

**Table 3:** Mean OHIP-G 49, mean OHIP-G 14 and mean COHIP 19 scores and subscores grouped by potential influencing factors ‘high-quality treatment outcome’, ‘greatly improved’ and ‘habit of smoking’ Totale OHIP-G-49-, OHIP-G-14- und COHIP-19-Mittelwerte sowie der Mittelwerte der Subskalen, gruppiert nach potenziell beeinflussenden Faktoren ‘high-quality treatment outcome’, ‘greatly improved’ und ‘habit of smoking’

	High-quality treatment outcome Yes/No	*p*-value^a^	Greatly improved Yes/No	*p*-value^a^	Habit of smoking Yes/No	*p*-value^a^
*OHIP dimensions*	(*n* = 177/*n* = 36)	–	(*n* = 110/*n* = 103)	–	(*n* = 31/*n* = 182)	–
Functional limitations	3.04/3.22	0.724	2.95/3.19	0.501	*4.61/2.79*	*0.001*
Physical pain	2.12/1.61	0.075	1.99/2.09	0.697	1.97/2.07	0.738
Psychological disability	2.48/2.25	0.609	2.30/2.58	0.433	2.97/2.49	0.445
Psychological discomfort	0.51/0.33	0.126	0.41/0.55	0.151	0.58/0.47	0.440
Physiological disability	2.87/3.25	0.558	2.71/3.17	0.330	3.48/2.84	0.452
Social disability	0.60/0.44	0.412	0.52/0.63	0.500	0.84/0.54	0.279
Handicap	1.07/0.78	0.249	1.00/1.04	0.872	1.26/0.99	0.530
OHIP-G 49 total score	12.68/11.89	0.628	11.88/13.26	0.346	15.71/12.19	0.156
OHIP-G 14 total score	3.06/2.69	0.506	2.85/3.16	0.520	3.87/2.96	0.245
*COHIP dimensions*	(*n* = 92/*n* = 24)	–	(*n* = 51/*n* = 65)	–	–	–
Social/emotional, school and self-image	2.97/2.54	0.443	3.18/2.65	0.249		
Functional well-being	0.91/1.35	0.292	1.16/0.88	0.267		
Oral health	2.53/2.78	0.552	2.74/2.46	0.370		
COHIP—total score	6.41/6.58	0.874	7.04/5.98	0.172	–	–

*n* sample size, *OHIP* Oral Health Impact Profile, *COHIP* Child Oral Health Impact Profile

^a^Welch–Satterthwaite t‑test, values in italics represent statistical significance at 5%

Looking at patient-related influencing factors of OHRQoL, smokers reported significantly more impairment with regard to functional limitations than nonsmokers (4.61 vs. 2.79; *p* = 0.001). Their mean OHIP-G 49 score was also higher (15.71 vs. 12.19; *p* = 0.156; Table 3). There was no detectable influencing effect of study participants’ self-reported BMI on OHRQoL in the present patient sample.

Patients with an initial malocclusion according to KIG groups A, B and especially P reported comparably high impairment of the post-orthodontic OHRQoL (OHIP-G 49 total scores for A [*n* = 5] and B [*n* = 7] 17.00; for P [*n* = 11] 20.91). Looking at potential influencing factors within the KIG group P, a high total PAR score at the beginning of treatment was detected (29.00). In contrast, the final total PAR score and the PAR score reduction showed successful orthodontic treatment for both males and females (3.09 PAR points and 88.88% reduction). Within the 11 patients who initially classified for KIG-group P, 6 male patients most likely accounted for the above-mentioned high OHIP scores, compared to the mean OHIP score for all patients: While their PAR score reduction rate was even higher than that mentioned above (90.92%), their OHIP G‑49 total score was 28.17 (vs. 12.20 for female patients who classified for KIG group P).

#### COHIP

The mean COHIP score was 6.52 (inverted score 69.48; ± 4.07). Sex-related differences in COHIP scores were opposite to the trend mentioned above: female patients reported higher impairment than male patients (total score 6.99 vs. 5.84; *p* = 0.099; Table 2). Analyzing the data with regard to gender-specific differences, it was noticeable that fewer female patients finished with a ‘high-quality treatment outcome’ than males (total PAR score of 5 or less; 78.79% for females and 81.63% for males, respectively). Initial and final mean PAR scores did not differ greatly between the two sexes, neither did the percentages with regard to their orthodontic provider and treatment duration, nor the corresponding age distribution.

When looking at treatment- and occlusion-related aspects as potential influencing factors with regard to patients’ OHRQoL after orthodontic treatment, regardless of gender-specific deviations, no significant differences in COHIP sub- and total scores were observed about the PAR score reduction rate (Fig. 2) or the final PAR score in relation to the definition ‘high-quality treatment result’ (Table 3).

Patients, initially falling into the KIG groups B, M and especially O and P, reported about more impaired OHRQoL than patients with other types of malocclusion (COHIP total scores for B [*n* = 4] 6.75, for M [*n* = 11] 7.00, for O [*n* = 2] 12.00 and P [*n* = 19] 8.32). Because the KIG group P had been noteworthy with regard to OHIP scores as well, we looked at data from the corresponding 19 patients in detail. The 7 male patients within this group had a very high total PAR score at the beginning of their treatment (31.43) with corresponding high PAR scores for the PAR subgroups like ‘upper and lower anterior segments’, ‘overjet’ and ‘buccal segments’. The treatment of female patients within this group (*n* = 12) showed a lower PAR score reduction rate compared to males (79.03% vs. 88.70%). Post-orthodontic COHIP total score was 9.17 for females, compared to 6.86 for male patients within the KIG group P. No difference could be observed with regard to the orthodontic provider for these 19 patients.

### Patient-reported oral health

#### Oral hygiene

Patient-reported oral hygiene behavior was good regarding the frequency of tooth brushing per day as well as the duration of tooth brushing. The majority of patients brushed their teeth ‘usually twice a day’ (86% of all patients who filled out the OHIP-based questionnaire and 80% who filled out the COHIP-based questionnaire) for 2 min or more (75% of all patients who filled out the OHIP-based questionnaire and 81% who filled out the COHIP-based questionnaire).

Almost 60% of all patients who filled out the OHIP-based questionnaire and 54.0% who filled out the COHIP-based one used a manual toothbrush. All study participants used toothpaste with fluoride. The use of a mouthwash was part of the daily oral hygiene routine for many patients as well (50.5% for OHIP-based data and 50.4% for COHIP-based data).

#### Dental awareness, self-efficacy, and controlling conviction

**Fig. 3:**
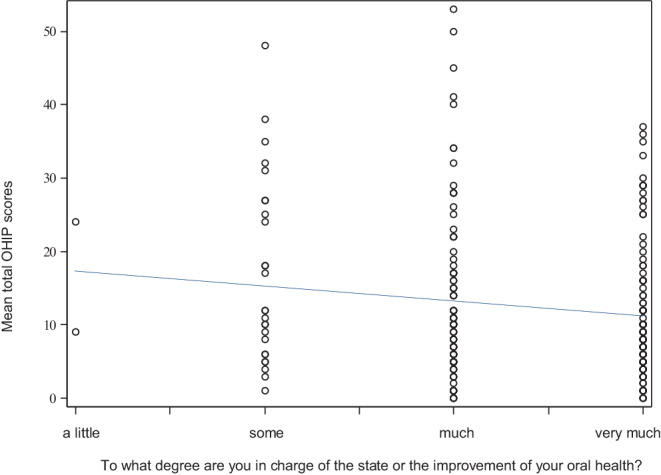
Linear regression analysis for the variables mean OHIP score and self-efficacy/locus of control (Question: To what degree are you in charge of the state or the improvement of your oral health?) Pearson’s correlation coefficients −0.134. *OHIP* Oral Health Impact Profile, *COHIP* Child Oral Health Impact Profile Lineare Regressionsanalyse für die Variablen OHIP-Mittelwert und orale Selbstwirksamkeit (Frage: Wie viel kann man selbst tun, um die Gesundheit seiner Zähne zu erhalten oder zu verbessern?) Pearsons Korrelationskoeffizient −0,134. *OHIP* Oral Health Impact Profile, *COHIP* Child Oral Health Impact Profile

Of all study participants who filled out the OHIP-based questionnaire, 87.4% considered themselves responsible for their own oral health. Only 2 participants believed in a rather external locus of control concerning their oral health. Figure 3 shows a trend that mean total OHIP scores were lower for those who strongly believed they were in charge of their own oral health, i.e., who believed in self-efficacy, than for those who did not (*p* = 0.142).

Furthermore, 57.7% of the above-mentioned 222 patients stated to visit their general dentist regularly, and another 27.5% occasionally. In addition, 81.5% stated to visit a general dentist for control check-ups whom they have known for years.

Of the study participants who filled out the OHIP-based questionnaire, 91.0% rated their oral condition to be either “very good” or “good”; this was also true for 90.1% of all patients who filled out the COHIP-based questionnaire.

Esthetic issues were the initial reason to start orthodontic treatment for 55.4% of those participants who filled out the OHIP-based questionnaire, 17.6% started orthodontics because of problems with their temporomandibular joints and 12.6% reported chewing difficulties as a reason for orthodontic counselling.

## Discussion

This multicenter cohort study focused on the quality of orthodontic care in a German convenience sample. While part 1 addressed treatment effectiveness according to occlusal outcome measures [1], the present part 2 dealt with cross-sectional data about patient-reported outcomes after orthodontic treatment. As a major part of these patient-reported outcomes, OHRQoL was evaluated using the German version of the COHIP and OHIP questionnaires through an iPad-based procedure. This process was not time-consuming for study participants, although the number of items per questionnaire was rather large. Mean levels of OHRQoL among study participants after orthodontic treatment were good and the average degree of impairment was low. Yet, because of the cross-sectional character of this study, pre-orthodontic patient-reported outcome values of study participants could not be obtained. This hindered the direct attribution of changes in OHRQoL to orthodontic interventions. Statements about the genuine effect of orthodontics on patient-reported outcomes cannot be made according to the present data. Nonetheless, taking the post-orthodontic OHIP-G 14 score of 3.09 in our study into account, it was comparably low with regard to national as well as international study populations [4, 15, 16]. The 6th German Oral Health Study used the OHIP-G 5 version to evaluate OHRQoL and found a mean score of 1.3. For our data, the OHIP-G 5 score was comparably low, being 1.4. However, the cited study sample comprised youths who were 8–9 years old and not adolescents or adults older than 16 years after orthodontic treatment as in our ‘OHIP study group’ [34].

The association between finished orthodontic treatments and OHRQoL is highly interesting and crucial to look at in the context of good quality of care. Silvola et al. conducted a large cohort study with 1885 Finnish adults and found that whenever study participants had been treated orthodontically, they reported better OHRQoL [5]. Zheng et al. described significant changes in OHRQoL throughout orthodontic treatment—depending on the initial malocclusion and treatment stage—in their Chinese population of adolescents and young adults and found final mean OHIP-14 scores between 2.98 and 3.23. In addition, a cohort of adult study participants who had undergone combined orthodontic–orthognathic surgery showed a mean OHIP-14 score of 4.1 at the end of their treatment. Moreover, the Finnish authors found a correlation between high initial PAR scores and more impaired OHRQoL [11]. In a systematic review, Mandava et al. came to the conclusion that fixed orthodontic treatment might improve OHRQoL and self-esteem in children as well as OHRQoL in adolescents and adults [35]. A potentially beneficial effect of orthodontic treatments with regard to OHRQoL has been shown by other authors as well [36–39].

To our knowledge, there is only very limited data available on COHIP scores *after* orthodontic treatment, in contrast to evidence about such scores *prior to* orthodontic treatment [6, 7, 16, 40].

Analyzing our data regarding potential influencing factors of OHRQoL after orthodontic treatment, the results showed a nonsignificant trend towards lower mean OHIP-G 49 and OHIP-G 14 scores for study participants whose treatments classified for the PAR category “greatly improved” (i.e., PAR score change of at least 22 points). While a variety of authors agree that higher initial levels of OHRQoL are associated with specific initial malocclusion traits like a compromised overjet [7, 40–42] or severe initial malocclusion in general [4, 43, 44], evidence is scarce about the correlation of patients’ OHRQoL after orthodontics and the degree of malocclusion changes throughout treatment [10, 45]. Further and longitudinal research is needed in order to fully understand this crucial association.

Looking for other influencing factors of OHRQoL after orthodontics, there seemed to be sex-related differences. Of the patients who filled out the COHIP questionnaire, i.e., study participants younger than 16 years, girls showed more impairment of oral health-related quality of life compared to boys after orthodontic treatment. Especially the dimensions of social and emotional well-being might be more impaired in girls than in boys during puberty, a time span of major life changes [44]. Sun et al. found similar results in their group of 15-year-old study participants. Yet, they emphasized that OHRQoL should be seen as a dynamic construct with numerous potential influencing factors that might change over the years [46]. For adult patients, several researchers reported such gender-dependent differences for OHRQoL, namely a tendency to a more impaired well-being for females than for males [4, 5]. Contrary to this, our study participants who were older than 16 years and who filled out the OHIP-based questionnaire, showed a reverse sex-related influence after orthodontic treatment: males reported slightly higher impact of their OHRQoL than females. The reasons for this outcome could not be fully identified. On average, orthodontic treatments lasted longer for males than females which might have caused perceived impairment [47].

Within our study population, OHRQoL was more impaired for smokers. Smoking is harmful—not only for general health, but also for oral health in particular. Oral cancer, periodontal disease, tooth loss or staining might be oral conditions resulting from excessive tobacco use [48, 49]. However, research on the association between OHRQoL and smoking is scarce [50–52]. Results of this multicenter study highlight the correlation between an impaired OHRQoL—the self-perceived impact of oral conditions—and smoking. The dimension ‘functional limitations’ within the OHIP-G 49 construct proved to be significantly compromised for orthodontically treated smokers. The respective dimension contains questions about the self-perception of tooth staining, bad breath or taste impairments. It is not surprising that these aspects of oral health showed significantly more impairment for smokers. Yet, because ‘smoking’ is not a topic of great interest within orthodontic research or within orthodontic treatments and the accompanying consultation sessions as such, it should be kept in mind while advising our patients about oral health-promoting ways of life, especially when treating adults.

Patients’ BMI had no influence on OHRQoL within the current study population. Due to incomplete data, the effect of patients’ socioeconomic status could not be analyzed, implicating a potential for bias.

Patient-reported oral hygiene behavior as well as the reported self-efficacy and dental awareness were good, especially compared to national cohorts [34, 53]. After orthodontic treatment, study participants brushed their teeth frequently and with adequate tools—according to their own reports. Almost 90% of the patients who filled out the OHIP-based questionnaire believed that they were in charge of their oral health. Apparently, they exhibited an internal control orientation. Interestingly, these patients showed a lower OHIP score compared to the patients who did not feel responsible for their oral health. According to the locus of control theory, people with an internal locus of control believe that they are responsible for their destinies [54]. In dentistry and specifically in orthodontics, this locus of control theory has been thought to help determine and enhance patients’ adherence. Yet, a linear correlation between an internal locus of control and patients’ ability to adjust to specific situations (like the insertion of orthodontic appliances) has been hard to prove in relevant literature [22–24]. Furthermore, almost 60% of the participants reported to visit their general dentist regularly. Up to 91% of all study participants found their oral condition either ‘very good’ or ‘good’ after orthodontics. These results from our national cohort highlight that orthodontically treated patients seem to be aware of their oral health and the possibilities to promote it.

There are several limitations of this multicenter study. A major drawback is the missing longitudinal patient-reported data. Thus, one can only judge the current, *post*-orthodontic state and cannot directly relate this to the malocclusion *prior* to orthodontics and/or the orthodontic treatment. Nevertheless, careful associations between the psychological and physical well-being of orthodontically treated patients and specific patient- and treatment-related factors might be highlighted and compared to existing longitudinal and cross-sectional study results as it has been done within this manuscript [55]. Yet, it has to be stressed that the cross-sectional study design does not allow any robust statement about potential effects of orthodontic treatment and generalizability is limited. Selection and nonresponder bias could not be completely ruled out. Although a strict screening of all potential study participants was mandatory as mentioned above, the study center selection as such had not been random. Thus, this sample can be seen as a German convenience sample and might not be representative for every orthodontic practice or department in Germany. External validity is compromised. In addition, another limitation of this study is that neither an a priori sample size calculation based on OHRQoL nor an alpha adjustment because of multiple testing was performed. The PAR score and its dynamics throughout treatment were the primary endpoints and sample size considerations were based on part 1 [1], while OHRQoL was a secondary outcome. Nonetheless, with regards to data from relevant literature, the current sample size seemed to be large enough to depict potential aspects about patient-reported outcomes in relation to PAR score dynamics within this cohort. In addition and already mentioned above, not all potentially confounding factors, for example, socioeconomic status have been taken into account in the present analyses.

## Conclusion

Posttreatment oral health-related quality of life (OHRQoL) proved to be on a good level and was rather unimpaired in this German cohort. Furthermore, self-reported oral hygiene behavior and oral health beliefs stood for a good health awareness. Patients’ gender as well as the habit of smoking seemed to affect OHRQoL after orthodontic treatment.

## Supplementary Information

Supplementary Questionnaire 1

Supplementary Questionnaire 2
