# Supplementary material for: Quality of orthodontic care—A multicenter cohort study in a German convenience sample: Part 2: Cross-sectional patient-reported outcomes after orthodontic treatment
Source: J Orofac Orthop. 2024 Apr 26;86(6):412–23. doi: 10.1007/s00056-024-00528-z (PMC12540623; doi:10.1007/s00056-024-00528-z)
Supplement: Supplementary file 1 — Supplementary Questionnaire 1 [file 56_2024_528_MOESM1_ESM.pdf]

Sehr geehrte Damen und Herren,

die Poliklinik für Kieferorthopädie der Uniklinik Köln führt gemeinsam mit verschiedenen Standorten in Deutschland eine multizentrische Untersuchung hinsichtlich der Zahngesundheit und der Behandlungsqualität von kieferorthopädischen Behandlungen durch.

Ihre Mitarbeit ist freiwillig. Die wissenschaftliche Aussagekraft der Untersuchung hängt aber entscheidend von der Mitarbeit aller ausgewählten Personen ab.

Bitte beantworten Sie daher diesen Fragebogen sorgfältig und eigenständig. Wenn Sie Fragen haben, können Sie sich jederzeit an unsere(n) Mitarbeiter/in wenden.

Vielen Dank für Ihre Mitarbeit!

Probanden Nr: Standortkürzel (K/Gie/BadE/Pu/Es/Aa/Ber)/ Modell-Nr (xxxx/xx) / „A“ oder „E“

Fragen für Probanden, die ≥ 16 Jahre alt sind

1. Wenn Sie an Ihre Zähne denken, wie ist der Zustand Ihrer Zähne?

- □ Sehr gut
- □ Gut
- □ Zufriedenstellend
- □ Weniger Gut
- □ Schlecht

2. Wie viel kann man selbst tun, um die Gesundheit seiner Zähne zu erhalten oder zu verbessern?

- □ Sehr Viel
- □ Viel
- □ Einiges
- □ Wenig
- □ Nichts

3. Wie oft putzen Sie sich gewöhnlich die Zähne?

- □ 3-mal täglich und häufiger
- □ Normalerweise 2-mal täglich
- □ Normalerweise 1-mal täglich
- □ Mehrmals die Woche
- □ 1-mal die Woche
- □ Seltener als 1-mal die Woche
- □ Eigentlich nie

4. Wann putzen Sie gewöhnlich die Zähne? *(Sie können auch mehreres ankreuzen!)*

- □ Nach dem Aufstehen, **vor** dem Frühstück
- □ **Nach** dem Frühstück
- □ **Nach** dem Mittagessen
- □ **Nach** dem Abendessen
- □ **Nach** Zwischenmahlzeiten
- □ **Bevor** ich ins Bett gehe
- □ **Verschieden**- wenn ich gerade daran denke

5.Wie lange putzen Sie sich die Zähne? *(Bitte versuchen Sie, in Minuten oder Sekunden zu schätzen!)*

- □ Ca. 30 Sekunden
- □ Ca. 1 Minute
- □ Ca. 1 ½ Minuten
- □ Ca. 2 Minuten
- □ Ca. 3 Minuten
- □ Länger als 3 Minuten

6.Weche Mittel benutzen Sie zur Mundpflege? *(Sie können auch mehreres ankreuzen!)*

- □ Zahnbürste
- □ Elektrische Zahnbürste
- □ Zahnpasta
- □ Zahnseide
- □ Zahnhölzer, -stocher, -stäbchen
- □ Zahnzwischenraumbürstchen
- □ Munddusche
- □ Mundwasser, Mundspüllösung
- □ Zuckerfreie Kaugummis
- □ Sonstige Pflegemittel
- □ Keine Pflegemittel

7. Ist bei Ihnen jemals vom Zahnarzt eine Behandlung des Zahnfleisches (Parodontitis-Behandlung) durchgeführt worden?

- □ Ja
- □ Nein

8. Gehen Sie zum Allgemeinzahnarzt (gemeint ist <u>nicht</u> der Kieferorthopäde) nur, wenn Sie Schmerzen oder Beschwerden haben, oder gehen Sie manchmal auch zur <u>Kontrolle</u>?

- □ Ich gehe **regelmäßig** zur Kontrolle
- □ Ich gehe auch **manchmal** zur Kontrolle
- □ Ich gehe **nur**, wenn ich Schmerzen / Beschwerden habe
- □ Ich gehe **nicht** zum Zahnarzt

9. Haben Sie einen Zahnarzt, den Sie als <u>Ihren</u> Zahnarzt bezeichnen würden?

- □ Ich bin seit Jahren bei demselben Zahnarzt in Behandlung
- □ Ich habe keinen festen Zahnarzt, ich wechsle öfter den Zahnarzt
- □ Ich habe im Moment keinen Zahnarzt
- □ Ich war noch nie beim Zahnarzt

10. Warum haben Sie Sich entschieden, eine kieferorthopädische Behandlung durchführen zu lassen?

- □ Beschwerden beim Kauen
- □ Probleme mit dem Kiefergelenk
- □ Ästhetische Gründe
- □ Auf Rat eines Bekannten / Verwandten

11. Rauchen Sie zur Zeit Zigaretten? Wenn ja, wie viele und seit wann?

- □ Ja, ___ Zigarette (n) am Tag seit ___ Jahren
- □ Nein

Hatten Sie in den vergangenen Wochen aufgrund von Problemen mit Ihren Zähnen und der kieferorthopädischen Behandlung….

	sehr oft	oft	ab und zu	kaum	nie
12. ….Schwierigkeiten beim Kauen von Nahrungsmitteln?	□	□	□	□	□
13. ….Schwierigkeiten, bestimmte Worte auszusprechen?	□	□	□	□	□
14. ….das Gefühl, dass ein Zahn nicht gesund aussieht?	□	□	□	□	□
15. ….das Gefühl, dass Ihr Aussehen beeinträchtigt wurde?	□	□	□	□	□
16. ….das Gefühl, Sie hätten Mundgeruch?	□	□	□	□	□
17. ….das Gefühl, Ihr Geschmackssinn war beeinträchtigt?	□	□	□	□	□
18. ….Speisereste zwischen Ihren Zähnen festgestellt?	□	□	□	□	□
19. ….das Gefühl, Ihre Verdauung war gestört?	□	□	□	□	□
20…..das Gefühl, dass Ihr Zahnersatz					

schlecht sitzt? □ □ □ □ □

Hatten Sie in den vergangenen Wochen ….

	sehr oft	oft	ab und zu	kaum	nie
21. ….Schmerzen im Mundbereich?	□	□	□	□	□
22. ….einen wunden, entzündeten Kiefer bzw. Mund?	□	□	□	□	□
23. ….Kopfschmerzen aufgrund von Problemen mit Ihren Zähnen, Mund-Bereich oder in Zusammenhang mit der kieferorthopädischen Behandlung?	□	□	□	□	□
24. ….empfindliche Zähne, z.B. bei heißen/kalten Speisen bzw. Getränken?	□	□	□	□	□
25. ….Zahnschmerzen?	□	□	□	□	□
26. ….Schmerzen am Zahnfleisch?	□	□	□	□	□

War es Ihnen in den vergangenen Wochen aufgrund von Problemen mit Ihren Zähnen und der kieferorthopädischen Behandlung, ….

	sehr oft	oft	ab und zu	kaum	nie
27. …. unangenehm, bestimmte Nahrungsmittel zu essen?	□	□	□	□	□

Hatten Sie in den vergangenen Wochen ….

	sehr oft	oft	ab und zu	kaum	nie
28. ….wunde Stellen in Ihrem Mund?	□	□	□	□	□
29. **Hat ihr** Zahnersatz im vergangenen Monat unangenehm gedrückt?	□	□	□	□	□

30. ….**Haben Sie sich im vergangenen Monat** Sorgen gemacht? ☐ ☐ ☐ ☐ ☐

Hatten Sie in den vergangenen Wochen ….

	sehr oft	oft	ab und zu	kaum	nie
31. ….ein Gefühl der Unsicherheit in Zusammenhang mit Ihren Zähnen bzw. Ihrem Mund?	☐	☐	☐	☐	☐
32. ….das Gefühl, dass es Ihnen ganz schlecht geht aufgrund Ihrer Zähnen bzw. Ihrem Mund?	☐	☐	☐	☐	☐
33. ….das Gefühl, dass Sie sich unbehaglich/unwohl wegen des Aussehens Ihrer Zähne gefühlt haben?	☐	☐	☐	☐	☐
34. ….ein Gefühl, dass Sie angespannt waren aufgrund von Problemen mit Ihren Zähnen, im Mundbereich oder mit Ihrer kieferorthopädischen Behandlung?	☐	☐	☐	☐	☐

Ist es Ihnen in den vergangenen Wochen aufgrund von Problemen mit Ihren Zähnen und der kieferorthopädischen Behandlung vorgekommen, ….

	sehr oft	oft	ab und zu	kaum	nie
35. ….dass Sie nur undeutlich sprechen konnten?	☐	☐	☐	☐	☐
36. ….dass andere Leute bestimmte Worte von Ihnen missverstanden haben?	☐	☐	☐	☐	☐
37. ….dass Sie den Eindruck hatten, Ihr Essen war geschmacklich weniger gut?	☐	☐	☐	☐	☐
38. ….dass Sie Ihre Zähne nicht richtig putzen konnten?	☐	☐	☐	☐	☐
39. ….dass Sie auf bestimmte Speisen verzichtet haben?	☐	☐	☐	☐	☐

40. ….dass Ihre Ernährung unbefriedigend gewesen ist? □ □ □ □ □

41. ….dass Sie mit Ihrem Zahnersatz nicht essen konnten? □ □ □ □ □

42. ….dass Sie das Lächeln vermieden haben? □ □ □ □ □

43. ….dass Sie Mahlzeiten unterbrechen mussten? □ □ □ □ □

44. ….dass Ihr Schlaf unterbrochen wurde? □ □ □ □ □

45. ….dass Sie sehr verärgert waren? □ □ □ □ □

Hatten Sie in den vergangenen Wochen ….

	sehr oft	oft	ab und zu	kaum	nie
46. ….Schwierigkeiten zu entspannen in Zusammenhang mit Ihren Zähnen bzw. Ihrem Mund?	□	□	□	□	□
47. ….das Gefühl, dass Sie bedrückt/depressiv waren aufgrund von Problemen mit Ihren Zähnen bzw. Ihrem Mund?	□	□	□	□	□
48. ….ein Gefühl sich schlechter konzentrieren zu können aufgrund von Problemen mit Ihren Zähnen bzw. Ihrem Mund?	□	□	□	□	□
49. ….das Gefühl ein wenig verlegen zu sein aufgrund von Problemen mit Ihren Zähnen oder Ihrem Mund?	□	□	□	□	□

Ist es Ihnen in den vergangenen Wochen aufgrund von Problemen mit Ihren Zähnen und der kieferorthopädischen Behandlung vorgekommen,….

	sehr oft	oft	ab und zu	kaum	nie

50. ….dass Sie es vermieden haben, außer Haus
zu gehen aufgrund von Problemen mit Ihren
Zähnen oder Ihrem Mund? ◻ ◻ ◻ ◻ ◻

51. ….dass Sie weniger nachsichtig im Umgang
mit Ihrem Ehepartner oder Ihrer Familie waren
aufgrund von Problemen mit Ihren
Zähnen oder Ihrem Mund? ◻ ◻ ◻ ◻ ◻

52. ….allgemein Schwierigkeiten zu haben, mit anderen Menschen
zurechtzukommen aufgrund von Problemen mit Ihren
Zähnen oder Ihrem Mund? ◻ ◻ ◻ ◻ ◻

53. ….dass Sie eher reizbar anderen Menschen
gegenüber waren aufgrund von Problemen mit Ihren
Zähnen oder Ihrem Mund? ◻ ◻ ◻ ◻ ◻

54. ….dass es Ihnen schwergefallen ist,
Ihren alltäglichen Beschäftigungen
aufgrund von Problemen mit Ihren
Zähnen oder Ihrem Mund nachzugehen? ◻ ◻ ◻ ◻ ◻

Hatten Sie in den vergangenen Wochen ….

	sehr oft	oft	ab und zu	kaum	nie
55. ….den Eindruck, das sich Ihre Allgemeingesundheit v5rschlechtert hat aufgrund von Problemen mit Ihren Zähnen oder Ihrem Mund?	◻	◻	◻	◻	◻
56. ….finanzielle Einbußen aufgrund von Problemen mit Ihren Z5hnen oder Ihrem Mund?	◻	◻	◻	◻	◻
57. ….das Gefühl, dass Sie die Gesellschaft anderer Menschen nicht so recht genießen konnten aufgrund von Problemen mit Ihren Zähnen oder Ihrem Mund?	◻	◻	◻	◻	◻
58. ….das Gefühl, dass Ihr Leben ganz allgemein weniger zufriedenstellend war aufgrund von Problemen mit Ihren Zähnen oder Ihrem Mund?	◻	◻	◻	◻	◻
59. ….das Gefühl vollkommen unfähig zu sein, um etwas zu tun aufgrund von Problemen mit Ihren Zähnen oder Ihrem Mund?	◻	◻	◻	◻	◻

60. ….das Gefühl, dass Sie bei Ihrer Arbeit nicht so leistungsfähig waren aufgrund von Problemen mit Ihren Zähnen oder Ihrem Mund? ◻ ◻ ◻ ◻ ◻

Haben Sie in den vergangenen Wochen ….

	sehr oft	oft	ab und zu	kaum	nie
61. ….es vermieden, mit anderen Personen gemeinsam zu essen aufgrund von Problemen mit Ihren Zähnen oder Ihrem Mund?	◻	◻	◻	◻	◻
62. …das Gefühl gehabt, dass es länger gedauert hat eine Mahlzeit zu beenden aufgrund von Problemen mit Ihren Zähnen oder Ihrem Mund?	◻	◻	◻	◻	◻

Hatten Sie in den vergangenen Wochen ….

	sehr oft	oft	ab und zu	kaum	nie
63. ….ein störendes Geräusch im Kiefergelenk?	◻	◻	◻	◻	◻
64. ….einen unangenehm trockenen Mund?	◻	◻	◻	◻	◻

Wann wurden Sie geboren?

Welches Geschlecht haben Sie?

Wie viel Kilogramm wiegen Sie ohne Bekleidung?

Und wie groß sind Sie ohne Schuhe?

Welchen Schulabschluss haben Sie? (Volksschul-/Hauptschulabschluss/Mittlere Reife, Realschulabschluss/Abschluss 10. Klasse/Fachholschulreife/Abitur/Anderen Schulabschluss/Noch keinen Schulabschluss, zur Zeit noch in Schule/Nichts davon, keinen

Schulabschluss)

Welche der folgenden aufgaben trifft auf Ihre derzeitige Situation zu? (Zur Zeit nicht oder nicht mehr berufstätig/Teilzeit oder stundenweise berufstätig/Voll berufstätig/vorübergehende Freistellung (z.B. Elternzeit)/Vorzeitig in Rente, pensioniert/Arbeitslos gemeldet/Hausfrau-/Mann/in Ausbildung)

Vielen Dank für die Beantwortung der Fragen!
