# Supplementary material for: Quality of orthodontic care—A multicenter cohort study in a German convenience sample: Part 2: Cross-sectional patient-reported outcomes after orthodontic treatment
Source: J Orofac Orthop. 2024 Apr 26;86(6):412–23. doi: 10.1007/s00056-024-00528-z (PMC12540623; doi:10.1007/s00056-024-00528-z)
Supplement: Supplementary file 2 — Supplementary Questionnaire 2 [file 56_2024_528_MOESM2_ESM.pdf]

Liebe Jugendliche,

die Poliklinik für Kieferorthopädie der Uniklinik Köln führt zusammen mit einigen anderen Studienzentren/Städten eine Untersuchung in Zusammenhang mit kieferorthopädischen Behandlungen durch. Dabei befragen wir in ganz Deutschland Jugendliche in Deinem Alter, aber auch Erwachsene.

Deine Mitarbeit ist freiwillig. Es ist aber von entscheidender Bedeutung, dass gerade Du bei dieser Studie mitmachst- Du wurdest zufällig als Vertreter für viele Jugendliche in Deinem Alter ausgewählt. Du trägst also mit Deinen Angaben einen Mosaikstein zum Gesamtbild aller dieser Jugendlichen bei- ohne Dich wäre das Bild unvollständig!

Bitte beantworte daher den Fragbogen sorgfältig und eigenständig, wenn nötig mit Deiner Mutter oder Deinem Vater. Wenn Du Fragen hast, kannst Du dich jederzeit an unsere(n) Mitarbeiter/in wenden.

Vielen Dank für Deine Mitarbeit!

Probanden Nr: Standortkürzel (K/Gie/BadE/Pu/Es/Aa/Ber)/ Modell-Nr (xxxx/xx) / „A“ oder „E“

Fragebogen für Patienten, die ≥11 ≤ 15 Jahre alt sind

Wie oft in den letzten drei Monaten ….

	niemals	fast niemals	manchmal	ziemlich oft	fast immer
1. ... hattest Du Zahnschmerzen?	◻	◻	◻	◻	◻
2. ... hattest Du verfärbte Zähne oder Flecken auf Deinen Zähnen?	◻	◻	◻	◻	◻
3. ... hast Du festgestellt, dass Deine Zähne schief sind oder Du Lücken zwischen den Zähnen hast?	◻	◻	◻	◻	◻

4. ... hattest Du einen schlechten Atem? □ □ □ □ □

5. ... hattest Du Zahnfleischbluten? □ □ □ □ □

6. ... hattest Du Schwierigkeiten, wegen Deiner Zähne,
Deines Mundes oder Gesichtes Essen zu essen, das Du
gerne gegessen hättest? □ □ □ □ □

7. ... hattest du wegen Deiner Zähne, Deines Mundes oder
Gesichtes Schwierigkeiten, zu schlafen? □ □ □ □ □

8. ... fiel es dir schwer, bestimmte
Wörter auszusprechen aufgrund Deiner Zähne,
Deines Mundes oder Gesichtes? □ □ □ □ □

9. ... hattest Du wegen Deiner Zähne,
Deines Mundes oder Gesichtes Schwierigkeiten,
Deine Zähne sauber zu halten? □ □ □ □ □

10. ... warst Du traurig oder betrübt wegen Deiner Zähne,
Deines Mundes oder Gesichtes? □ □ □ □ □

11. ... warst Du wegen Deiner Zähne, Deines Mundes oder
Gesichtes aufgewühlt und unbehaglich? □ □ □ □ □

	niemals	fast niemals	manchmal	ziemlich oft	fast immer

12. ... hast Du wegen Deiner Zähne,
Deines Mundes oder Gesichtes vermieden,
mit anderen Kindern zu lächeln oder
zu lachen? □ □ □ □ □

13. ... hast Du das Gefühl gehabt,
dass Du anders aussiehst aufgrund Deiner Zähne,
Deines Mundes oder Gesichtes? □ □ □ □ □

14. ... warst Du besorgt darüber,
was andere Leute über Deine Zähne,
Deinen Mund oder Dein Gesicht denken? □ □ □ □ □

15. ... wurdest Du von anderen Kindern gehänselt,
schikaniert oder beschimpft aufgrund Deiner Zähne, Deines
Mundes oder Gesichtes? □ □ □ □ □

16. ... hast Du die Schule wegen Deiner Zähne, Deines
Mundes oder Gesichtes versäumt? □ □ □ □ □

17. ... wolltest Du vor der Klasse nichts sagen oder auch nicht laut vorlesen aufgrund Deiner Zähne, Deines Mundes oder Gesichtes? □ □ □ □ □

Wie oft in den letzten drei Monaten ….

	niemals	fast niemals	manchmal	ziemlich oft	fast immer
18. ... warst Du selbstsicher wegen Deiner Zähne, Deines Mundes oder Gesichtes?	□	□	□	□	□
19. ... hattest Du das Gefühl wegen Deiner Zähne, Deines Mundes oder Gesichtes gut auszusehen?	□	□	□	□	□

20. Wenn Du an Deine Zähne denkst, wie ist der Zustand Deiner Zähne?

- □ Sehr gut
- □ Gut
- □ Mittelmäßig
- □ Eher schlecht
- □ Schlecht

21. Wie oft putzt Du Dir gewöhnlich die Zähne?

- □ 3-mal täglich und häufiger
- □ Normalerweise 2-mal täglich
- □ Normalerweise 1-mal täglich
- □ Mehrmals die Woche
- □ 1-mal die Woche
- □ Seltener als 1-mal die Woche
- □ Eigentlich nie

23. Wie lange putzt Du Dir die Zähne? *(Bitte versuche, in Minuten oder Sekunden zu schätzen!)*

- ◻ Ca. 30 Sekunden
- ◻ Ca. 1 Minute
- ◻ Ca. 1 ½ Minuten
- ◻ Ca. 2 Minuten
- ◻ Ca. 3 Minuten
- ◻ Länger als 3 Minuten

24. Welche Mittel benutzt Du zur Mundpflege? *(Du kannst auch mehreres ankreuzen!)*

- ◻ Zahnbürste
- ◻ Elektrische Zahnbürste
- ◻ Zahnpasta
- ◻ Zahnseide
- ◻ Zahnhölzer, -stocher, -stäbchen
- ◻ Zahnzwischenraumbürstchen
- ◻ Munddusche
- ◻ Mundwasser, Mundspüllösung
- ◻ Zuckerfreie Kaugummis
- ◻ Sonstige Pflegemittel
- ◻ Keine Pflegemittel

Wann wurdest Du geboren?

Dein Geschlecht?

In die wievielte Klasse gehst Du?
(5.-13. Klasse; Ausbildung)

Was für eine Schule besuchst Du?
(Gesamtschule, Hauptschule, Realschule, Gymnasium, Förderschule, Ausbildung, Berufsschule)

Vielen Dank für die Beantwortung der Fragen!
